# Insulin Deprivation Decreases Caspase-Dependent Apoptotic Signaling in Cultured Rat Sertoli Cells

**DOI:** 10.1155/2013/970370

**Published:** 2013-10-09

**Authors:** T. R. Dias, L. Rato, A. D. Martins, V. L. Simões, T. T. Jesus, M. G. Alves, P. F. Oliveira

**Affiliations:** CICS-UBI, Health Sciences Research Centre, Faculty of Health Sciences, University of Beira Interior, Avenue Infante D. Henrique, 6201-506 Covilhã, Portugal

## Abstract

Insulin is essential for the regulation of glucose homeostasis. Insulin dysfunction occurs in several pathologies, such as diabetes mellitus, which is associated with fertility problems. Somatic Sertoli cells (SCs) not only metabolize glucose to lactate, which is the central energy source used by developing germ cells, but also determine the germ cell population size. If a deregulation in SCs apoptosis occurs, it will affect germ cells, compromising spermatogenesis. As SCs apoptotic signaling is a hormonally regulated process, we hypothesized that the lack of insulin could lead to alterations in apoptotic signaling. Therefore, we examined the effect of insulin deprivation on several markers of apoptotic signaling in cultured rat SCs. We determined mRNA and protein expression of apoptotic markers as well as caspase-3 activity. SCs cultured in insulin deprivation demonstrated a significant decrease on mRNA levels of p53, Bax, caspase-9, and caspase-3 followed by a significant increase of Bax and decrease of caspase-9 protein levels relatively to the control. Caspase-3 activity was also decreased in SCs cultured in insulin deprivation conditions. Our results show that insulin deprivation decreases caspase-dependent apoptotic signaling in cultured rat SCs evidencing a possible mechanism by which lack of insulin can affect spermatogenesis and fertility.

## 1. Introduction

Sertoli cells (SCs) are highly polarized epithelial cells [1] that play a central role in the functional development of the testis and hence in the expression of the male phenotype [2, 3]. They actively metabolize glucose to lactate [4], which is then used by developing germ cells as the main substrate for ATP production [5, 6]. Each SC has a fixed capacity for the number of germ cells that it can support [7], though this capacity varies between species. So, the number of SCs will determine the number of germ cells that can be supported through spermatogenesis [7, 8] by regulating germ cells apoptotic rates [9]. As SCs can only accommodate the differentiation of a finite number of germ cells [7], a deregulation in SCs apoptosis could affect spermatogenesis, resulting in a lower fertility or even in infertility. 

Apoptosis is a result of a complex network of signaling pathways, which allows the organism to tightly control cell numbers and tissue size and to protect itself from rogue cells that threaten homeostasis [10, 11]. During stress signaling, p53 can be accumulated in cells and,when activated, initiates a cascade of events [12]. p53 regulates Bax [13], which is a proapoptotic protein of the Bcl-2 family that targets the mitochondria causing the release of apoptotic signaling molecules [14]. Otherwise, Bcl-2, an antiapoptotic member of the Bcl-2 family, maintains the mitochondria membrane potential preventing the release of those apoptotic signaling molecules [15, 16]. Thus, the  ratio Bax/Bcl-2 determines the response to a death signal [17]. If apoptotic signaling molecules reach the cytosol, they can recruit caspase-9 [18, 19]. Once activated through cleavage, caspase-9 activates the downstream effector caspase-3 that irreversibly causes apoptosis [20]. Several studies have shown that key proteins involved in apoptotic signaling interact with and have effects on cellular energy metabolism and homeostasis [21]. 

SC glucose metabolism regulation is crucial for normal spermatogenesis and fertility [22] and the hormonal control of SC metabolism regulates spermatogenesis [4]. Several hormones such as 5*α*-dihydrotestosterone (DHT) and 17*β*-estradiol (E2) [23, 24] are reported as metabolic modulators of SCs. Recently, it has been reported that insulin is also crucial for a normal SC metabolism [25]. Insulin dysfunction has been associated to a widely known metabolic disorder, diabetes mellitus (DM) [26]. Type 1 diabetes mellitus (T1DM) is characterized by the autoimmune destruction of insulin-producing pancreatic islet beta cells [27, 28] in genetically susceptible individuals, resulting in the total lack of insulin. Several studies using animal models strongly suggest that T1DM impairs male fertility [29, 30]; however, the involved mechanisms are not fully understood. Insulin deprivation is known to induce several important metabolic alterations in cultured SCs [25]. Alterations of cell metabolism, particularly in glucose metabolism, are closely related with the apoptotic process [21]. Accordingly, we hypothesized that these metabolic alterations induced by insulin deprivation could lead to abnormal apoptosis in SCs and thus trigger the spermatogenesis and fertility problems ascribed to T1DM individuals. Herein we analyze the effect of insulin deprivation on cultured rat SCs apoptotic signaling. For that we evaluated mRNA and protein expression levels of key points of the mitochondrial apoptotic pathway: p53, Bcl-2, Bax, caspase-9, and caspase-3. The Bax/Bcl-2 ratio was also evaluated. Finally, caspase-3 activity was determined as an endpoint marker of apoptosis. 

## 2. Methods

### 2.1. Chemicals

M-MLV RT and random primers were purchased from Invitrogen (Carlsbad, CA, USA). dNTPs were purchased from GE Healthcare (Buckinghamshire, UK). Taq DNA polymerase was purchased from Fermentas Life Science (Ontario, Canada). Caspase-3 substrate was purchased from Calbiochem (Darmstadt, Germany). Polyclonal antibodies were obtained from Santa Cruz Biotechnology (Heidelberg, Germany). TRIzol reagent and other drugs were obtained from Sigma-Aldrich (St. Louis, MO, USA).

### 2.2. Animals

The present study used five 3-month-old male Wistar rats. They were obtained from vivarium sources and maintained on *ad libitum* food and water in a constant temperature (20 ± 2°C) room on a 12-hour cycle of artificial lighting. Rats were fed with a standard chow diet (4RF21 certificate, Mucedola, Italy). The animals were anesthetized and sacrificed by cervical displacement. All the animal experiments were performed according to the “Guide for the Care and Use of Laboratory Animals”; published by the US National Institutes of Health (NIH publication no. 85-23, revised 1996) and the rules for the care and handling of laboratory animals (directive 86/609/EEC).

### 2.3. Sertoli Cell Culture

After animals were sacrificed, the testis was immediately excised in aseptic conditions and washed two times in ice cold HBSS containing 10000 U/mL of penicillin, 10 mg/mL streptomycin, and 25 *μ*g/mL amphotericin B (pH 7.4). SCs were isolated by slight modifications of the method previously described [31, 32]. SC culture purity was assessed by the immunoperoxidase technique. Briefly, cells were grown on cell culture dishes, incubated overnight at 4°C with primary polyclonal antibody and labelled streptavidin-biotin method using an ExtrAvidin-Peroxidase Staining Kit, giving a brown coloration to the SCs after reaction with diaminobenzidine. The cell nuclei were then stained with haematoxylin. Negative-control incubations were executed using PBS instead of primary antibody. Specific protein markers, anti-mullerian hormone (AMH) and vimentin, were used to assess the purity of rat SCs cultures as described elsewhere [33]. After 96 h, cultures were examined by phase contrast microscopy and only the cultures with cell contaminants below 5% were used.

### 2.4. Experimental Groups

SCs were allowed to grow until reaching 90%–95% of confluence, and after being fully washed the medium was replaced by serum-free medium (supplement DMEM : F12 1 : 1, pH 7.4). SCs cultures were divided into two groups: in the control group the SCs medium was supplemented with insulin-transferrin-sodium selenite (10 mg/mL–5.5 mg/mL–5 *μ*g/mL, resp.) while the insulin-deprived group was supplemented with transferrin-sodium selenite (5.5 mg/mL–5 *μ*g/mL, resp.). After 96 hours of treatment, cells were detached from the flask using a Trypsin-EDTA solution and collected for RNA and protein extraction. SCs number was determined with a hemacytometer. Trypan blue exclusion assays were used to determine the percent survival of the SCs after their isolation. Viability average was 85%–90%.

### 2.5. Polymerase Chain Reaction

PCR was performed to analyze p53, Bcl-2, Bax, caspase 9, and caspase 3 mRNA expression as described by Rato and collaborators [27]. Optimal annealing temperature is shown in Table 1. mRNA expression was normalized with 18S gene expression and expressed as fold variation (induction/reduction) versus the control group.

### 2.6. Western Blotting

Total proteins were isolated from rat SCs using RIPAS buffer (1x PBS, 1% NP-40, 0.5% sodium deoxycholate, 0.1% SDS, 1 mM PMSF, supplemented with 1% protease inhibitor cocktail, aprotinin, and 100 mM sodium orthovanadate). Protein concentration was determined by BioRad (Hemel Hempstead, UK) Bradford microassay according to the manufacturer's instructions. Protein samples (50 *μ*g) were mixed with sample buffer (1.5% Tris, 20% glycerol, 4.1% SDS, 2% *β*-mercaptoethanol, 0.02% bromophenol blue, pH 6.8) in a final volume of 20 *μ*L, sonicated for 10 minutes at 4°C and denatured for 15 minutes at 55°C. Proteins were fractionated in 20% polyacrylamide gels and electrophoresis was carried out for 75 min. The separated proteins were then transferred to previously activated polyvinylidene difluoride membranes and then blocked for 90 min in a 5% nonfat milk solution at room temperature. Afterwards, the membranes were incubated overnight at 4°C with rabbit anti-Bax (1 : 1000, Cell Signaling Technology), rabbit anti-Bcl-2 (1 : 2500, Cell Signaling Technology), rabbit anti-p53 (1 : 200, Cell Signaling Technology), or rabbit anti-caspase 9 IgG (1 : 2000, BD Pharmingen) primary antibodies. Mouse anti-*α*-tubulin (1 : 5000, Sigma Aldrich) was used as the protein loading control. The immunoreactive proteins were detected separately and visualized with goat anti-rabbit IgG-AP (1 : 5000, Santa Cruz Biotechnology) or goat anti-mouse IgG-AP (1 : 5000, Santa Cruz Biotechnology). Membranes were reacted with ECF (GE, Healthcare) and read with the BioRad FX-Pro-plus (Bio-Rad, UK). Densities from each band were obtained with BIO-PROFIL Bio-1D Software from Quantity One (Vilber Lourmat, Marne-la-Vallée, France) according to standard methods [34]. The band density attained was divided by the corresponding *α*-tubulin band intensities and expressed in fold variation (induction/reduction) versus the control group. 

### 2.7. Caspase-3 Activity

Caspase-3 activity was spectrophotometrically assessed by determining the cleavage of the respective colorimetric substrate as previously described [35]. Briefly, proteins (25 *μ*g) were incubated with the assay buffer (25 mM HEPES, pH 7.5, 0.1% CHAPS, 10% sucrose, and 10 mM DTT) and 100 *μ*M of caspase-3 substrate (Ac-DEVD-pNA) for 2 h at 37°C. The caspase-3 activity was determined by detection of the chromophore p-nitroaniline, measured at 405 nm in a spectrophotometer. The method was calibrated with known concentrations of p-nitroaniline. The attained activities were expressed in percentage versus the control group.

### 2.8. Statistical Analysis

The statistical significance of mRNA and protein expression levels and caspase-3 activity among the experimental groups was assessed by two-way ANOVA, followed by Bonferroni posttest. All experimental data are shown as mean ± SEM (*n* = 5 for each condition). Statistical analysis was performed using GraphPad Prism 5 (GraphPad Software, San Diego, CA, USA). *P* < 0.05 was considered significant.

## 3. Results

### 3.1. mRNA Expression Levels of p53 and Bax Are Decreased in SCs Cultured in Insulin Deprivation Conditions Although Bax Protein Levels Are Increased

**Figure 1:**
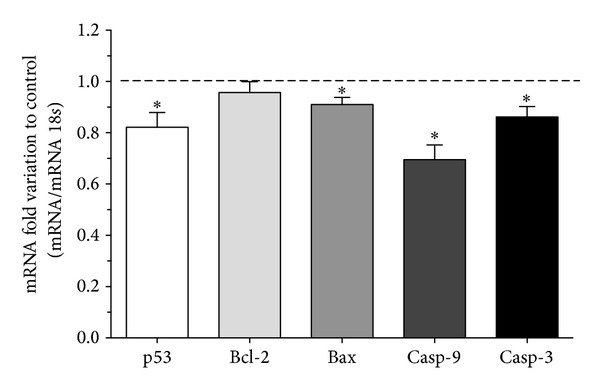
Effect of insulin deprivation on p53, Bcl-2, Bax, caspase-9, and caspase-3 mRNA levels in rat Sertoli cells. The figure shows pooled data of independent experiments, indicating the fold variation of mRNA levels found in rat SCs cultured in insulin deprivation conditions when compared with the control condition (dashed line). For each sample, the obtained band intensity was divided by the respective 18S band intensity, to obtain the relative abundance in each experimental condition. Results are expressed as mean ± SEM (*n* = 5 for each condition). Significantly different results (*P* < 0.05) are indicated: *relatively to control.

**Figure 2:**
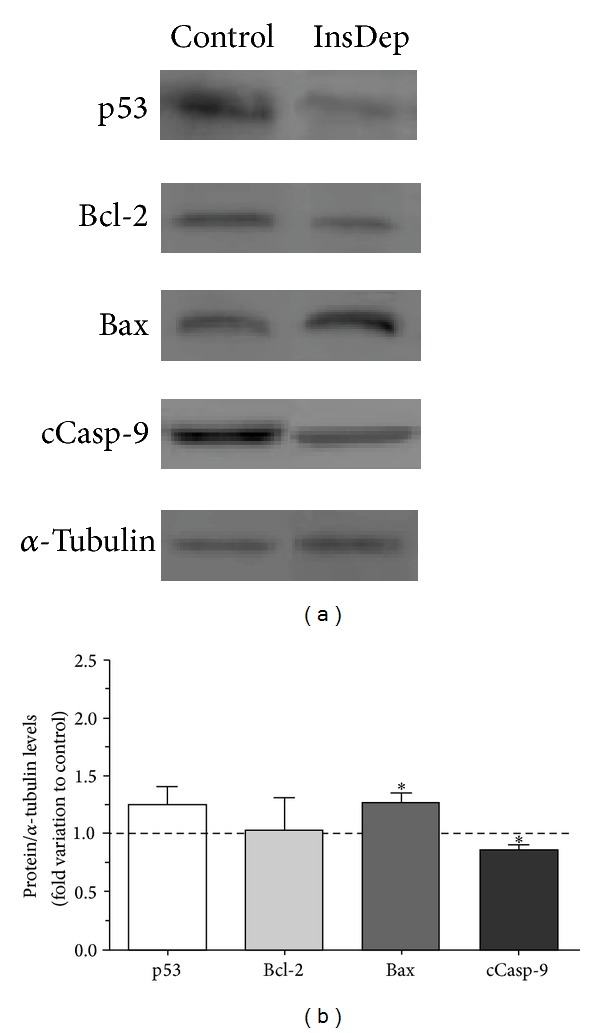
Effect of insulin deprivation on p53, Bcl-2, Bax, and cleaved caspase-9 protein levels in rat Sertoli cells. (a) shows representative western blot experiment. (b) displays pooled data of independent experiments, indicating the fold variation of protein levels found in rat SCs cultured in insulin deprivation conditions (InsDep) when compared with the control condition (dashed line). Results are expressed as mean ± SEM (*n* = 5 for each condition). Significantly different results (*P* < 0.05) are indicated: *relatively to control.

**Figure 3:**
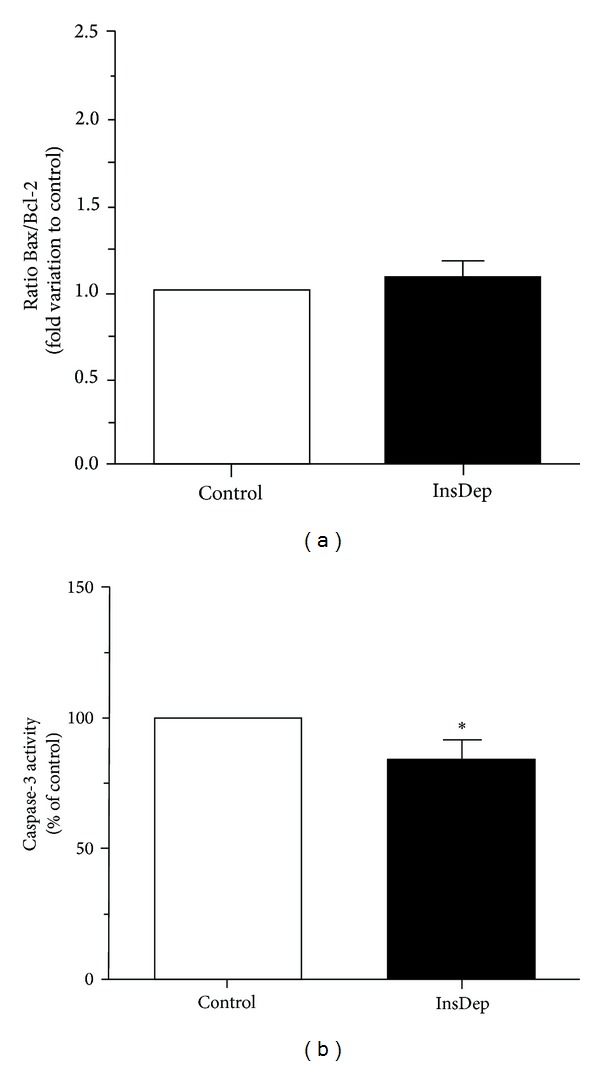
Effect of insulin deprivation on Bax/Bcl-2 ratio (a) and caspase-3 activity (b). Results are presented as pooled data of independent experiments, indicating the percentage variation found in rat SCs cultured in insulin deprivation (InsDep) conditions when compared with the control condition. Results are expressed as mean ± SEM (*n* = 5 for each condition). Significantly different results (*P* < 0,05) are indicated: *relatively to control.

SCs viability was not altered when the cells were cultured in insulin deprivation conditions as evaluated by trypan blue exclusion (data not shown), but we hypothesized that SCs apoptotic signaling could be altered when these cells are under insulin deprivation conditions. In response to a myriad of stress signals, the p53 protein is activated and thereafter, depending on the tissue type and the extent of the cellular damage, triggers adequate cellular response including apoptosis [36]. SCs mRNA expression levels of p53 decreased to 0.82 ± 0.06-fold (Figure 1) after the 96 hours of insulin deprivation. This significant decrease in p53 mRNA levels observed in SCs cultured in insulin deprivation conditions was not accompanied by any alteration in p53 protein levels (Figure 2(b)). The p53 gene product is known to be an upstream regulator of the Bax gene [13]. p53 binds to the Bax gene promoter and directly transactivates the transcription of this proapoptotic gene [37]. Thus, we evaluated the mRNA and protein levels of Bax in both SCs cultured in control and insulin deprivation conditions. Our results show that Bax mRNA levels are significantly decreased in 0.91 ± 0.03-fold variation to the control in SCs cultured in insulin deprivation conditions (Figure 1). However, Bax protein levels are significantly increased in SCs cultured in insulin deprivation conditions to 1.27 ± 0.08-fold variation relatively to the SCs cultured in control conditions (Figure 2(b)). Bax is a proapoptotic protein, member of the Bcl-2 family, which facilitates caspases activation [38]. Otherwise, the B-cell lymphoma/leukemia-2 gene, commonly known by Bcl-2 [39], is an antiapoptotic member of the Bcl-2 family that maintains the mitochondria membrane potential and prevents the release of apoptotic proteins [15, 16, 40]. Thus, we determined the mRNA and protein levels of Bcl-2 in SCs cultured in control and insulin deprivation conditions. Our results showed that SCs cultured in insulin deprivation conditions did not present any alteration in the mRNA expression levels of Bcl-2 (Figure 1). This is concomitant with the results obtained in protein expression levels, since Bcl-2 protein levels did not present differences when comparing SCs cultured in control and insulin deprivation conditions (Figure 2(b)). Finally, we evaluated the ratio of Bax/Bcl-2. This ratio is essential since it acts as a rheostat that determines the susceptibility to apoptosis in cells by regulating mitochondrial function [37]. Our results show that this ratio is not altered in SCs cultured in insulin deprivation conditions when compared with SCs from the control group (Figure 3(a)). 

### 3.2. Caspase-9 mRNA Levels and Cleaved Caspase-9 Protein Expression Are Decreased in SCs Cultured in Insulin Deprivation Conditions

Caspase proteins are cysteine proteases that act downstream of the Bcl-2 family by initiating cellular breakdown during apoptosis [41]. Caspase-9 is a cysteine aspartyl protease associated with the intrinsic or mitochondrial pathway of apoptosis [18, 42]. SCs cultured in insulin deprivation conditions presented a significant caspase-9 mRNA levels decrease to 0.70 ± 0.06-fold variation relatively to cells cultured in control conditions (Figure 1). The binding of procaspase-9 to apoptotic peptidase activating factor 1 (Apaf-1) leads to the autolytic cleavage of procaspase-9 [43], which allows the initiation of the caspases cascade [44]. Thus, we evaluated the protein levels of cleaved caspase-9. Our results demonstrated that insulin deprivation significantly decreased cleaved caspase-9 levels in SCs to 0.87 ± 0.04-fold variation relatively to SCs cultured in control conditions (Figure 2(b)). 

### 3.3. Insulin Deprivation Significantly Decreases Caspase-3 mRNA Levels and Activity in SCs

Once activated, caspase-9 cleaves and activates the effector caspase-3 which targets key regulatory and structural proteins for proteolysis to affect apoptosis [45]. The activation of caspase-3 turns the apoptotic pathway irreversible and thus we evaluated caspase-3 mRNA levels and caspase-3 activity by measuring the cleavage of a specific substrate and the release of a chromophore (p-nitroaniline). Following the significant decrease in both, caspase-9 mRNA levels and cleaved caspase-9 protein levels, in SCs cultured in insulin deprivation conditions, our results revealed that caspase-3 mRNA levels also decreased significantly in those cells to 0.86 ± 0.04-fold variation relatively to the control group (Figure 1). Thus, as expected, caspase-3 activity was also decreased to 83 ± 8.33% in SCs cultured in insulin deprivation conditions relatively to cells cultured in control conditions (Figure 3(b)). 

## 4. Discussion

In T1DM, there is an absolute lack of insulin and testicular biopsies of diabetic patients which revealed numerous abnormalities in SCs [46]. Nevertheless, the mechanisms underlying these malfunctions remain largely unknown [47]. Noteworthy, there are few reports versed on the mechanisms behind the infertility that is known to occur in individuals with T1DM. There is also a lack of literature concerning the insulin control of spermatogenesis [47, 48]. Recently, Oliveira and collaborators [24] described that human SCs cultured in insulin deprivation conditions presented altered glucose consumption, lactate secretion, and altered expression of metabolism-associated genes involved in lactate production and export as well as glucose uptake. Moreover, acetate production by SCs was also reported to be completely suppressed after only 6 hours of insulin deprivation [25]. These first reports concerning the effect of insulin deprivation in SCs, were focused in metabolism and metabolism-associated pathways. Nevertheless, alterations of cell metabolism, particularly in glucose metabolism, are closely related with the apoptotic process [21]. Indeed, mitochondria are the connection between apoptotic and metabolic signaling. They are the source of several proapoptotic proteins and remain the central organelle in the apoptotic pathway. Hence, following previously reported results that emphasized a severe effect of insulin deprivation in SCs metabolism, we hypothesized that the lack of insulin could actively modulate the regulation of pro- and/or antiapoptotic proteins.

The apoptotic pathway can be triggered in response to extracellular cues and internal insults [10]. We determined the effect of insulin deprivation on the expression of some apoptotic markers related to the apoptotic pathway in cultured rat SCs. In the apoptotic pathway, the tumor suppressor protein p53 is a key intervenient. When p53 is activated, it can stimulate the expression of proapoptotic factors, such as Bax [49]. Our results showed that both p53 and Bax mRNA levels are decreased in cells cultured in insulin deprivation conditions. Moreover, the slight increase in p53 protein levels was also followed by a significant increase on Bax protein levels in SCs cultured in a medium without insulin. This is in agreement with the fact that p53 is a well-known upstream regulator of the Bax gene. However, protein levels did not follow the tendency of mRNA gene expression. While p53 and Bax mRNA levels decreased, protein levels increased. As mRNA is eventually translated into protein, it is usually assumed that there is some sort of correlation between the levels of mRNA and protein [41]. But, there are several reasons for the poor correlations generally reported between the mRNA and protein levels that may not be mutually exclusive. First, there are many complicated and diverse postranscriptional mechanisms involved in turning mRNA into protein that are not yet sufficiently well defined to be able to compute protein levels directly from mRNA levels; second, proteins may differ substantially in their *in vivo* half-lives; third, there is a significant amount of error and noise in both protein and mRNA experiments that limit a clear correlation [50, 51]. So, despite the evaluation of mRNA and protein levels complement each other, the obtained results must be analyzed independently and not as correlated sources of information. Therefore, our results suggest that p53 and Bax are downregulated in SCs cultured under insulin deprivation conditions although only Bax protein levels are significantly increased. An explanation for Bax increasing and not p53 is that there is not a direct correlation between protein levels of p53 and Bax. For instance, the Bax promoter can be activated by wild-type but not mutant p53 [37] and increased expression of wild-type p53 and a low p53 mutation rate can occur in SCs cultured in insulin deprivation conditions, as it happens in other mammalian cells [37]. The activation of the pro-apoptotic factor Bax leads to the mitochondria permeabilization, which causes the release of several apoptotic factors from the intermembrane space that trigger the caspase-dependent death pathway, through the sequential activation of cleaved caspase-9 and the effector caspase-3 [52], or the caspase-independent death effectors, which translocate to the nucleus and contribute to chromatin condensation and chromatinolysis [53]. 

The activation of antiapoptotic proteins is often described as a critical point in the control of the apoptotic process. These antiapoptotic proteins, belonging to Bcl-2 family proteins, function as antagonists of the pore forming in the mitochondrial membrane interrupting the apoptotic process [54]. The manipulation of this Bcl-2 system is suggested to be of extreme importance in pathological conditions that induce apoptotic pathways dysfunction. Our results showed that Bcl-2 mRNA and protein expression levels were not altered suggesting that insulin does not regulate this system. However, the Bax/Bcl-2 ratio seems to be more important in determining the sensitivity to apoptotic stimuli than the expression levels of each protein individually [37]. Again, our results demonstrated that this ratio was not significantly altered in SCs cultured in insulin deprivation conditions evidencing that the increased protein levels of pro-apoptotic Bax detected in cells maintained in insulin deprivation conditions were compensated by antiapoptotic Bcl-2 so that Bax/Bcl-2 and consequently the apoptotic signaling are maintained.

The ratio Bax/Bcl-2 is not altered between the experimental groups suggesting that mitochondrial membrane integrity is preserved. Following the apoptotic cascade, we assessed caspase-9 and caspase-3 levels. We observed a significant downregulation of caspase-9 mRNA levels in SCs cultured in insulin deprivation conditions concomitant with the significant decrease detected in cleaved caspase-9 protein levels. Once activated, caspase-9 cleaves and activates the effector caspase-3. Caspase-3 is usually known as an endpoint for apoptosis, marking the nonreturning point of the apoptotic process [55]. Indeed, caspase-3 activity is widely used as a hallmark for apoptosis, as it allows a qualitative and a quantitative assessment of the apoptotic process [56]. As expected, caspase-3 mRNA levels and activity were significantly decreased in SCs cultured in insulin deprivation conditions. Thus, our results show that insulin deprivation decreases the caspase-dependent apoptotic signaling in cultured rat SCs. Since SCs are responsible for determining the number of germ cells that can be supported through spermatogenesis [7, 8], consequently an alteration in SCs apoptotic levels may be sufficient to alter germ cells population. However, SCs require a well functioning of glucose metabolism to provide the amount of energy necessary to germ cells [3, 22, 57]. Recent studies proved that insulin deprivation in SCs resulted in altered glucose consumption and a substantial decrease in lactate secretion [25]. Although cultured SCs in insulin deprivation conditions proved to adapt their glucose metabolism [25], they are not able to support the energy requirements of germ cells since lactate production, the major germ cells energy substrate, is significantly decreased. This emphasizes the importance of the regulation of SCs apoptosis as decreased apoptotic signaling in SCs under these conditions may alter spermatogenesis and fertility. This mechanism may explain the fertility problems and SCs abnormalities found in male with T1DM [29, 30, 46], which represents an *in vivo* situation characterized by the total lack of insulin. The experiments presented herein are a first step to elucidate the role of insulin in the apoptotic control of SCs. Taken together, our results suggest that T1DM associated fertility problems may be linked to a malfunctioning of metabolism and apoptotic signaling in SCs.

The role of insulin in the apoptotic pathways of SCs has not been investigated so far. Nevertheless, in other cellular systems, such as neurons [58], insulin deprivation has proved to induce apoptosis. Noteworthy, high levels of insulin were also associated with stimulation of the apoptotic pathways in beta cells [59] thus evidencing the importance of this hormone in the regulation of the apoptotic process. SCs are not an exception and our results point towards a possible role of insulin in the regulation of the caspase-dependent apoptotic pathway. This is in agreement with previously described results supporting that DHT and E2 have an antiapoptotic action in *in vitro* immature rat SCs [60]. Our results confirm that apoptosis is a hormonally regulated process. As the apoptotic events controlled by SCs are crucial for the normal development of spermatogenesis and several pathologies are associated with insulin dysfunction, it is imperative to fully disclose the mechanisms by which insulin exerts its control over SCs apoptosis. 

## Figures and Tables

**Table 1:** Oligonucleotides and cycling conditions for PCR amplification of p53, bcl-2, Bax, caspase 9, caspase 3, and 18S.

Gene	Primer sequences	AT	C	Size (bp)
p53	Forward—CTG CCC ACC ACA GCG ACA GG	59°C	35	471
	Reverse—AGG AGC CAG GCC GTC ACC AT			
Bcl-2	Forward—GGG CTA CGA GTC GGA TAC	53°C	35	64
	Reverse—AGG CTG GAA GGA GAA GAT G			
Bax	Forward—CGC GTG GTT GCC CTC TTC TAC TTT	59°C	35	124
	Reverse—CAA GCA GCC GCT CAC GGA GGA			
Caspase 9	Forward—TGC AGG GTA CGC CTT GTG CG	61°C	35	130
	Reverse—CCT GAT CCC GCC GAG ACC CA			
Caspase 3	Forward—AGG CCT GCC GAG GTA CAG AGC	61°C	35	255
	Reverse—CCG TGG CCA CCT TCC GCT TA			
18s	Forward—AAG ACG AAC CAG AGC GAA AG	56°C	25	149
	Reverse—GGC GGG TCA TGG GAA TAA			

AT: annealing temperature; C: number of cycles during exponential phase of amplification.

## Abbreviations

AMH: Anti-Mullerian hormone
Apaf-1: Apoptotic peptidase activating factor 1
ATP: Adenosine-5′-triphosphate
BTB: Blood-testisbarrier
DHT: 5*α*-dihydrotestosterone
DM: Diabetes mellitus
DMEM: Dulbecco's modified eagle medium
dNTPs: Deoxynucleotide triphosphates
E2: 17*β*-estradiol
EDTA: Ethylene diamine tetra acetic acid
F12: Ham's nutrient mixture F12
HBSS: Hank's balanced salts solution
ITS: Insulin-transferrin-sodium selenite
M-MLV RT: Moloney murine leukemia virus reverse transcriptase
RT-PCRL: Reverse transcriptase polymerase chain reaction
SC: Sertoli cell
T1DM: Type 1 diabetes mellitus.
